# The Effect of Ultrasound on the Functional Properties of Wheat Gluten

**DOI:** 10.3390/molecules16054231

**Published:** 2011-05-23

**Authors:** Haihua Zhang, Irakoze P. Claver, Ke-Xue Zhu, Huiming Zhou

**Affiliations:** 1 School of Food Science and Technology, Jiangnan University, Wuxi, Jiangsu 214122, China; 2 Département de TIAA, Institut Supérieur d’Agriculture, Université du Burundi, BP. 35 Gitega, Burundi

**Keywords:** wheat gluten, ultrasound, rheology, emulsion properties, foam properties

## Abstract

In this study, the effect of ultrasound on the foaming and emulsifying properties of wheat gluten as well as its electrophoretic and rheology properties were investigated. The foam capacity and foam stability of ultrasound treated wheat gluten proteins gradually increased as the treatment power increased, and were more pronounced at 100% power level. Excluding those of the raw wheat gluten, the lowest emulsifying capacity values and emulsifying stability were obtained with the samples ultrasound treated at 60% power level. In general, ultrasound treatment did not cause major changes on the protein electrophoretic patterns of gluten samples at the power levels used. Ultrasound affected the storage and the loss moduli with typical U-shape alteration.

## 1. Introduction

The use of ultrasound technology for the modification of various biotechnological processes in the food industry has attracted an astounding level of attention over the last decade due to its rapid and reagent-less influence. The effects of ultrasound modification in the food industry are mainly attributed to the acoustic cavitation phenomenon [1]. When ultrasound is applied to a liquid system, a series of compression and refraction waves are induced on the molecules of the liquid medium as the ultrasound passes through via the ultrasound propagation pathway [2]. When the power is high enough to ensure that the refraction cycle exceeds the attractive forces of the liquid molecules, cavitation bubbles form from gas nuclei existing within the fluid. These bubbles grow to a critical size over the period of a few cycles until they become unstable and collapse violently [3]. The implosion of cavitation bubbles leads to energy accumulations further generating extreme temperatures (5,000 K) and pressures (1,000 atm), which in turn produce high shear energy waves and turbulence in the cavitation zone. The combination of pressure, heat and turbulence affects the ultrasounded liquid [4]. This is one aspect of the acoustic cavitation phenomenon. Another aspect is the development of strong micro-streaming currents resulting from bubble size variation and subsequent collapse of bubbles. Commonly, strong micro-streaming currents are associated with high-velocity gradients and shear stresses that can alter the characteristics of the liquid system [5]. The third aspect of the acoustic cavitation phenomenon is the generation of highly reactive free radicals through breakage of water molecules [6]; these reactive free radicals can then react with and modify other molecules. Moreover, part of the acoustic energy can be absorbed as heat, inducing temperature increases in the liquid. In general, the basic principle of ultrasound modification is through the physical, mechanical and chemical result of acoustic cavitation and is a process involving the formation, growth and violent collapse of small bubbles in liquid as a result of acoustic pressure fluctuations. 

Wheat gluten is an economically important co-product of the wheat starch industry. The vital wheat gluten has been mainly used in the field of bakery [7]. The prospects for utilization of wheat gluten are expanding due to the availability of gluten on the market at a relatively low cost. However, the expanding utilization of wheat gluten in food and nonfood industries [8,9] has been limited by a general lack of some desirable functional properties, such as solubility, foaming and rheological properties. Efforts to improve the functional properties of wheat gluten are unfolding challenges. Methods, including physical [10,11], chemical [12,13] and enzymatic [14,15] ones, have been widely used, but little is known about the effect of ultrasound technology on the functional properties of cereal proteins, especially wheat gluten protein. In this paper, ultrasound technology was used to improve the functional properties of wheat gluten. Therefore, the objective of this study was to determine the effects of ultrasound treatment on the physical properties of wheat gluten and then additionally explore the potential usage of treated wheat gluten.

## 2. Results and Discussion

### 2.1. Temperature Changes in Ultrasound Treated Wheat Gluten Suspensions

**Figure 1:**
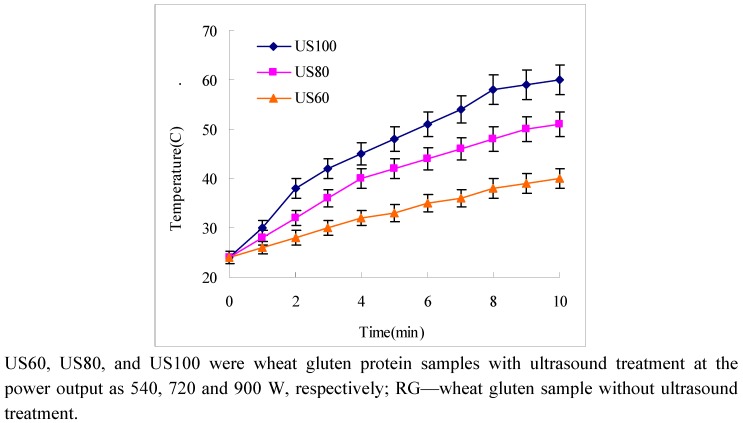
Temperature alteration of ultrasound wheat gluten suspensions as a function of time.

Part of the acoustic energy can be absorbed as heat; however, depending on the operating conditions and substrate, the temperature of wheat gluten suspension linearly increased as ultrasound treatment time was extended (Figure 1). The temperature increased as the ultrasound power increased. The highest temperature reached was 60 °C and was obtained by US100 for 10 min continous flow of ultrasonic energy. 

### 2.2. Foaming Properties of Ultrasound Treated Wheat Gluten

**Figure 2:**
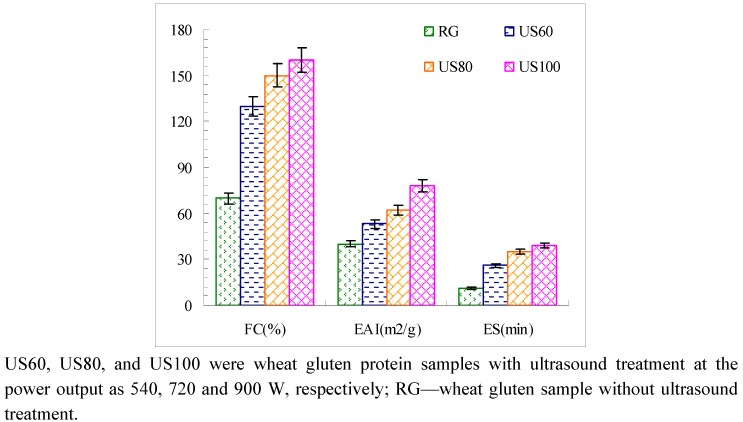
Foam capacity, Emulsifying properties, and Emulsion stability of ultrasound treated gluten protein samples.

**Figure 3:**
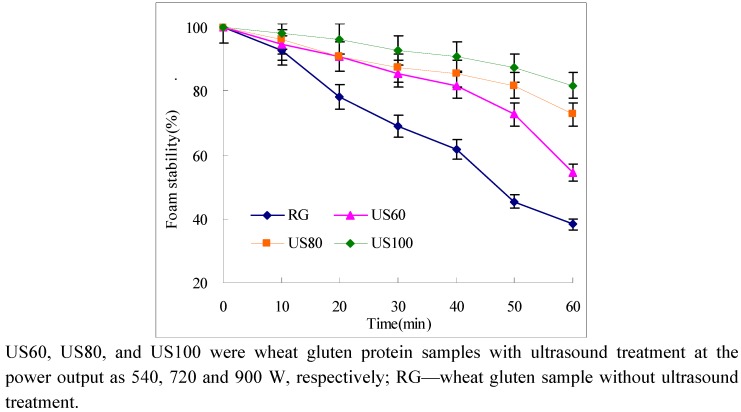
Foam stability of ultrasound treated gluten protein samples.

Proteins are good foaming agents, since they can diffuse to the air-water interface and thereby form a strong cohesive and elastic film by partial unfolding. Foaming capacity (FC) and foam stability (FS) of raw gluten and ultrasound treated gluten proteins dispersed in distilled water are presented in Figure 2 and Figure 3, respectively. Compared with RG, the foam capacity was improved to the highest value of 160% from 70% of RG at US100. A significant large increase in foam capacity with increasing ultrasound power level was observed for ultrasound-treated wheat gluten (Figure 2). The larger increases of foaming capacity observed might result from denaturation of wheat protein. Jambrak *et al*. [16] found that 20 kHz of ultrasound treatment for 15 min can cause denaturation of soybean protein concentrate and influence gelling. In our research, the condition of 20 kHz of ultrasound treatment for 10 min with contentious flow was used to treat wheat gluten. This condition may somehow cause denaturation of wheat gluten. It is commonly considered that denaturation could expose hydrophobic regions. For adsorption on the air/water interface molecules should contain hydrophobic regions [17].

The US60 retained 55% of the initial foam after 60 min of resting, while the raw gluten retained 38% of initial foam (Figure 3). Furthermore, the foam stability increased as ultrasound power increased, and the highest foam stability of 83% was observed in the US100. According to Lim and Barigou’s [18] report, protein-stabilized films rely on the viscoelastic properties of superficial “skin” to dissipate any local film stretching, and this mechanism relies on the immobilization. The superficial “skin” was a highly cohesive surface layer which was formed through interaction between neighboring protein molecules. Wheat protein is typically viscoelastic, and mainly composed of two kinds of proteins - glutenin and gliadin. Relying on inter/intra interaction of these two proteins, wheat gluten could form gluten net with air-holding, herein used for baking bread. Here, the interaction of unfold proteins might give an improvement of foam stability of ultrasound treated gluten.

### 2.3. Emulsifying Properties of Ultrasound Treated Wheat Gluten

Figure 2 shows the change in emulsifying properties of ultrasound treated wheat glutens. The EAI is a measure of the effectiveness of proteinaceous emulsifiers. Figure 2 shows that EAI and ES of ultrasound treated wheat glutens increased significantly as ultrasound power increased. It is suggested by previous studies that ultrasound generated emulsions are often more stable than those produced conventionally [19]. For example, stable emulsions of palm oil and β-lactoglobulinealginate complexes were produced, since ultrasound treatment was able to reduce the degree of droplet flocculation in these emulsions [20]. The decrease in the droplet size induced by ultrasound treatment was assumed to be the main reason for alterations in emulsifying properties of soy protein isolates [16].

### 2.4. SDS-PAGE Patterns of Wheat Gluten Proteins

**Figure 4:**
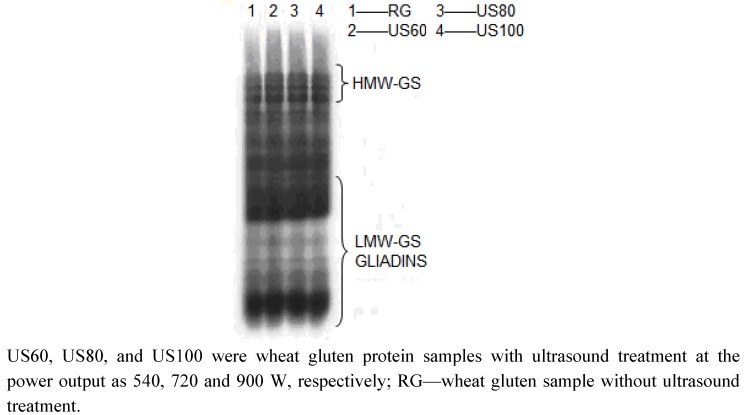
SDS-PAGE of ultrasound treated gluten protein samples.

SDS-PAGE patterns of ultrasound treated wheat glutens are presented in Figure 4, and the results showed that ultrasound treatment under condition we used did not induce major changes on the protein electrophoretic patterns of wheat gluten samples. When enough ultrasound power is inserted in the system, the molecular weight may be decreased causing a permanent viscosity reduction [21,22]. The inconsistence of our results with literature reports could be due to the differed origin of the protein and its physical properties. Wheat gluten protein is typically viscoelastic with strong mechanical tolerance. That is why ultrasound used had no major changes on wheat gluten protein.

### 2.5. Dynamic Rheological Properties of Ultrasound Treated Wheat Gluten

**Figure 5:**
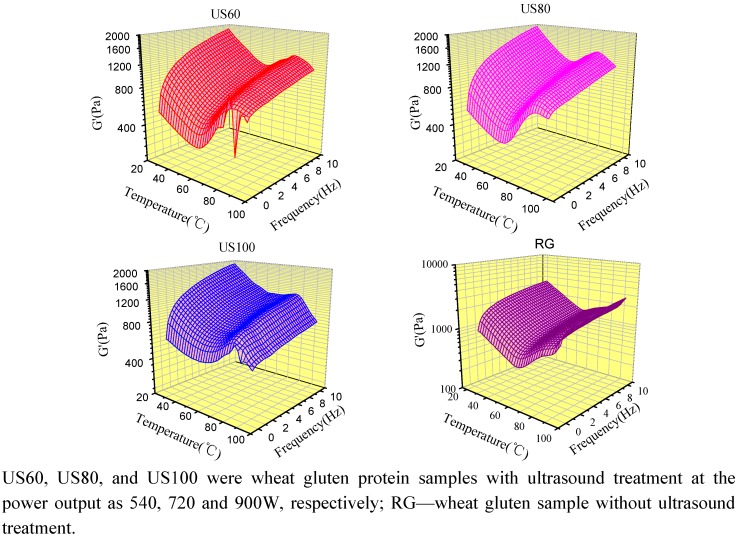
Storage module of ultrasound treated gluten samples as a function of temperature and frequency.

**Figure 6:**
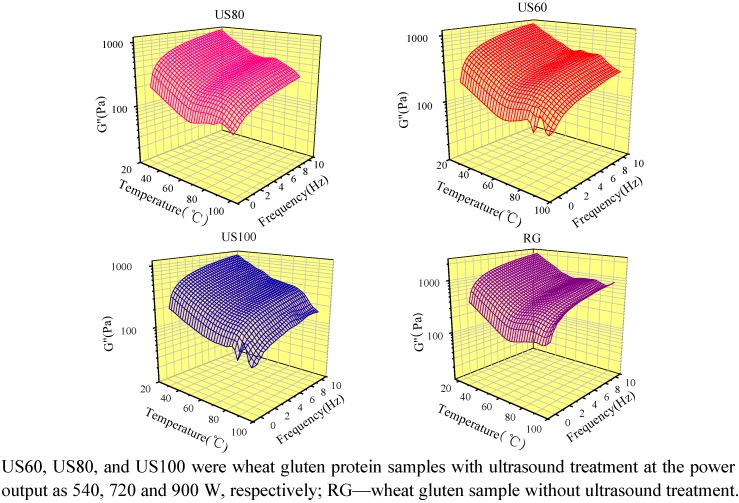
Loss module of ultrasound treated gluten samples as a function of temperature and frequency.

The effect of ultrasound treatments on the dynamic rheological properties of gluten was determined by method of oscillating temperature sweep at multiple frequency mode, and the storage (G') and loss (G'') moduli as a function of both temperature and frequency are respectively shown in three-dimensional Figure 5 and Figure 6. The main features of G' and G'', represent elastic and viscous character of the raw gluten and could be described as a wide U-shape sheet [23] when the temperature increased from 25 to 95 °C within the frequency range of 0.1–10 Hz. Under ultrasound treatments, the typical U-shape of gluten storage module (G') was affected. As shown in Figure 5 and Figure 6, rheological measurement of ultrasound treated gluten samples showed that a sharp and a mild transitions occurred in the dynamic storage moduli, G' and G'', at about 60 and 80 °C, respectively. The obvious changes after 80 °C showed that sharp decrease in G' and G'' of the ultrasound treated samples while their great increases in raw gluten were found. Generally, the G' and G'' of wheat gluten were decreased by ultrasound treatment. The lower G' and G'' of gluten after ultrasound treatments resulted from a decrease in the number of cross links between gluten polymers in the molecule, as ultrasound caused partial unfolding of wheat gluten protein which weakened the non-covalent bonding such as hydrogen bond between protein molecules [24]. 

## 3. Experimental

### 3.1. Materials and Chemicals

Wheat Gluten (protein 73% in wet base) was kindly donated by Lian Hua Ltd. Co. (Henan, China). 1-Anilino-8-naphthalene sulfonate (ANS) was purchased from Sigma Chemical Co. (Shanghai, China). All other chemicals used in the experiment were of analytical grade or the highest purity available from Chemical Co. (Shanghai, China). 

### 3.2. Ultrasound Treatment Protocol

Gluten (6 g) was dispersed into distilled water (100 mL) by magnetic stirring at 300 g for 10 min to form a homogeneous suspension. The thus formed suspension was sonicated with a high intensity ultrasound processor (Model KS-900; 20 kHz; 2 cm diameter horn; the maximum electric power of the ultrasound machine is 900 W; continuous mode; Haishu Kesheng Ultrasound Equipment Co., Ltd. Ningbo, China). The ultrasound probe was immersed 1.5 cm into the solution. The gluten solution was ultrasonicated at power settings of 540, 720 and 900 W (corresponding to 60, 80, and 100% of maximum power), respectively, for 10 min at 25 °C. Accordingly, the intensity of ultrasound was 10.8, 14.4 and 18.0 W·cm^−2^, and the specific heat was 0.43, 0.77 and 1.67 J·K^−1^·g^−1^, respectively. A thermocouple probe was immersed in the sample solution during the sonication to monitor the change of sample temperature. Each treatment was replicated three times. Thereafter, suspensions were freeze dried. The lyophilized ultrasound treated gluten samples were named as US60, US80, and US100, respectively, corresponding to the different power outputs of 60, 80 and 100%. A duplicate of the gluten suspension without ultrasound exposure was treated in the same way as the above gluten samples and was denoted as raw gluten control (RG). 

### 3.3. Foaming Capacity and Foam Stability

A 10% dispersion was mixed for 5 min using an FJ-200 homogenizer (Yueci Electronic Technology Co., Ltd. Shanghai, China). Foaming capacity (FC) was calculated as the % increase in volume of the protein dispersions upon mixing. Foam stability (FS) was estimated as the percent age of foam remaining after 60 min.

### 3.4. Emulsifying Properties

The emulsion activity index (EAI) of the samples was determined by the method stated by Wang *et al*. [12]. To prepare the emulsion, soybean oil (1.0 mL) and protein solution (0.2%, w/v, 3.0 mL) in 0.1 M phosphate buffer were shaken together and homogenized for 1 min at 12,000 rpm at 25 °C by an FJ-200 instrument. A sample of the emulsion (50 mL) was taken from the bottom of the container at different times and diluted with a 0.1% (w/v) sodium dodecyl sulfate (SDS) solution (5 mL). The absorbance of the diluted emulsion was determined at 500 nm. The emulsifying activity was determined from the absorbance measured immediately after emulsion formation. EAI was calculated as follows: 


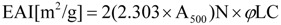


where A_500__nm_, absorbance; ϕ, volume fraction of dispersed phase; *L*, light path in metres; *C*, concentration of protein before the formation of the emulsion and *N*, diluted folds. The emulsion stability (ES) was estimated by measuring the time to reach half the initial turbidity of the emulsion. The ES was calculated as follows:


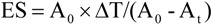


where A_0_, initial absorbance; A_t_, the absorbance determined at *t* minutes after emulsion formation; Δ*T*, time interval.

### 3.5. SDS-PAGE Analysis

Sodium dodecyl sulphate-polyacrylamide gel electrophoresis (SDS-PAGE) was conducted according to the method outlined by Singh and MacRitchie [6] using a 12% (v/w) acrylamide separating gel and 5% acrylamide stacking gel. Samples were prepared in Tris-glycine buffer (pH 8.8) containing 1.5% SDS and 0.2% β-mercaptoethanol. The gels were stained with Coomassie brilliant blue R-250.

### 3.6. Dynamic Rheological Measurements

A controlled stress rheometer (AR-G2 Rheometer, TA instrument Co. Branch Shanghai, China) was used to measure dynamic rheological properties of gluten samples and the method used was that presented by Georgopoulos *et al.* [26] with modifications. In order to ensure that all measurements were carried out within the linear viscoelastic regions, stress amplitude was selected ahead (data not shown). Based on these results, a stress amplitude of 5 Pa was chosen. A parallel plate measuring geometry was used (20 mm diameter), with a gap width of 2 mm. Sample pastes of 50% (w/v) were loaded onto the rheometer and allowed to equilibrate at the measuring temperature (25 ± 1 °C) for 5 min. Excess sample was trimmed off, and a thin layer of non-volatile silicone oil was applied to the exposed free edges to prevent moisture loss. Storage (G′) and loss (G′′) moduli were investigated with oscillate temperature sweep procedure with multiple frequencies in log-mode. Temperatures were varied from 25 to 95 °C in increments of 5 °C/min, and the frequency varied over the range of 0.1–10 Hz.

### 3.7. Statistic Analysis

Each determination was carried out on three separate samples and the results were shown in mean. The figures were edited by Origin 7.5 software (American Oringinlab Co. in Guangzhou, China). 

## 4. Conclusions

Protein emulsifying, foaming and dynamic rheological properties of gluten samples were significantly affected by ultrasound treatment. Emulsifying capacity was improved and the highest values were obtained with the samples treated at 900 W power level. The foam capacity and foam stability values of ultrasound treated gluten samples gradually increased with increasing power levels, which resulted from the exposure of hydrophobic regions for ultrasound caused partially unfolding of wheat protein. The typical U-shape of storage (G') and loss (G'') moduli were altered. When temperature over 60 °C, both storage (G') and loss (G'') moduli decreased under ultrasound treatment because of breakages of uncovalent bonds between gluten protein molecules. It was found that ultrasound power had no significant effect on the protein electrophoretic patterns of gluten samples. Based on these results, further studies would focus on the effect of ultrasound treatment on the structures of glidian and glutenin in order to give deeper insight into the effects of ultrasound modification on wheat gluten. 
